# Investigating the relationships between cognitive impairment score, cerebrospinal fluid measures and dementia diagnosis

**DOI:** 10.1002/alz.088365

**Published:** 2025-01-09

**Authors:** Zlatomira Georgieva Ilchovska, JeYoung Jung, Elizabeta Mukaetova‐Ladinska, Akram A. Hosseini

**Affiliations:** ^1^ University of Notingham, Nottingham UK; ^2^ University of Nottingham, Nottingham UK; ^3^ School of Psychology, University of Leicester, Leicester, Leicestershire UK; ^4^ Sir Peter Mansfield Imaging Centre, University of Nottingham, Nottingham UK; ^5^ Nottingham University Hospitals NHS Trust, Queens Medical Center, Nottingham UK; ^6^ Nottingham University Hospitals NHS Trust, Nottignham UK

## Abstract

**Background:**

Cerebrospinal fluid (CSF) dementia markers are sensitive to cognitive decline, particularly memory (Hosseini et al, 2023).

**Methods:**

We performed preliminary analyses on a neurodegenerative and non‐neurodegenerative patient dataset, collected as part of the CogNID study (IRAS reference: 250525, clinical trial reference: NCT03861884). We explore two questions, the first of which regards whether cognitive profile measured by the Addenbrooke’s cognitive examination (ACE‐R III) can predict diagnosis category, clustered into three groups: 1) Alzheimer’s Disease (AD; N=50), 2) non‐AD dementia conditions (e.g., frontotemporal dementia, Vascular Cognitive Impairment, Parkinson’s disease; N=46), and 3) a mixed, non‐dementia group serving as “control” (e.g., subjective cognitive impairment, post‐Covid‐19 brain fog; N=64). The second question asked whether, for a smaller subset of our participants with CSF dementia measures, there is any correlation between CSF dementia biomarkers and cognitive impairment. We explored these using regression analysis and Pearson’s correlation tests.

**Results:**

For our total sample with recorded ACE‐R III scores (N=160, M_age_=59.3, SD_age_=6.4), participants’ memory (t=‐2.31, p=.02) and language (t=2.16, p =.03) scores predicted their diagnosis category. Post‐hoc split‐data regression analyses revealed only a significantly lower memory score for the AD group compared to control group (t=‐2.482, p=.015). None of the other comparisons showed a significant difference after multiple‐comparisons corrections. For our second question, we selected a subsample of our data with recorded CSF measures (N=56). We assessed the CSF measures of total 181‐Tau, phospho‐Tau and Amyloidß_1‐42_ in these participants in relation to their memory, attention and visuospatial, obtained from the ACE‐R III scores. We found a significant negative correlation between 181‐phospho‐Tau (but not total Tau) and memory scores (r=‐.37, p=.005), indicating that higher 181‐phospho‐Tau measures were significantly associated with impaired memory performance.

**Conclusions:**

The preliminary analysis is limited by the high diversity of our neurodegenerative non‐AD group. A more detailed exploration of memory is crucial during diagnostic assessments of suspected AD.

**Disclosure and funding**: AAH is funded by the UK Medical Research Council (MR/T005580/1).

**Reference**: https://alz‐journals.onlinelibrary.wiley.com/doi/full/10.1002/alz.054530

